# Comparison of standard versus 90° rotation technique for LMA Flexible™ insertion: a randomized controlled trial

**DOI:** 10.1186/s12871-019-0773-z

**Published:** 2019-06-07

**Authors:** Bon-Wook Koo, Ah-Young Oh, Jung-Won Hwang, Hyo-Seok Na, Seong-Won Min

**Affiliations:** 1 Department of Anesthesiology and Pain Medicine, Seoul National University Bundang Hospital, 137-82 Gumi-ro, Bundang-gu, Sungnam-si, Gyeonggi-do 463-707 South Korea; 2 Department of Anesthesiology and Pain Medicine, Seoul National University College of Medicine, Seoul, South Korea; 3 Department of Anesthesiology and Pain Medicine, Boramae Hospital, Seoul, South Korea

**Keywords:** LMA flexible

## Abstract

**Background:**

Insertion of a flexible laryngeal mask airway (LMA Flexible) is known to be more difficult than that of a conventional laryngeal mask airway. The 90° rotation technique can improve the success rate with a conventional laryngeal mask airway but its effect with the LMA Flexible remains unknown. We assessed whether the 90° rotation technique increased the first-attempt success rate of LMA Flexible insertion versus the standard technique.

**Methods:**

In total, 129 female patients undergoing breast surgery were analyzed. The primary endpoint was success at the first attempt. The insertion time, number of trials, number of manipulations required, and oropharyngeal leak pressure were also evaluated. Heart rate and mean blood pressure were recorded 1 min before and 1 min after insertion. Blood staining on the LMA Flexible after removal and postoperative sore throat were checked.

**Results:**

The first-attempt success rates were comparable between the groups (93% vs. 98.3%, *P* = .20). The insertion time, number of trials and manipulations, hemodynamic variables, and complications, such as blood staining and sore throat, did not differ between the groups.

**Conclusions:**

The 90° rotation technique is a good alternative to the standard technique for insertion of the LMA Flexible.

**Trial registration:**

ClinicalTrials.gov (NCT03028896). It was registered retrospectively at Jan 19th, 2017.

## Background

The roles of supraglottic airways in anesthesia and airway management are increasing and are emphasized [1–4]. It is known that perioperative respiratory adverse events, such as laryngospasm, bronchospasm, sore throat, postoperative hoarse voice, and coughing, are decreased with the use of supraglottic airway compared to endotracheal tube [1–3]. The practice guidelines for management of the difficult airway by ASA also emphasize the role of supraglottic airway and recommends to always consider its use in the management of difficult airway [4].

The flexible laryngeal mask airway, LMA Flexible (Teleflex Co., Westmeath, Ireland), has a unique design, allowing the tube to be moved out of the surgical field without displacement of the cuff or loss of a seal. It also allows the tube complete flexibility and resistance to compression, such that the head and neck can be turned without dislodging the mask. The manufacturer recommends its use in head and neck surgery instead of tracheal intubation, such as bilateral myringotomy tubes, rhinoplasty, nasal sinus surgery, adenoidectomy, and tonsillectomy. Regarding the ease of insertion of the LMA Flexible, controversy exists. Some authors stated that it is more difficult to insert because the elasticity of its shaft makes it difficult to insert. They proposed tools to improve insertion of the LMA Flexible, such as a modified Magill forcep, a stylet, the Bosworth introducer, and the spatula introducer [5–8]. However, similar ease of insertion of the LMA Flexible is also reported [9]. 100% success rate of insertion of the LMA Flexible at the first attempt with the use of stadard insertion technique was also reported [10].

The 90° rotation technique was first described by Hwang et al. and involves the following steps: the entire cuff of the LMA is inserted inside the mouth, rotated counter-clockwise through 90° and advanced until the resistance of the hypopharynx is felt [11]. The use of this method is known to increase the success rate of insertion and decrease the incidence of blood staining of the LMA and sore throat compared to standard technique when inserting a Proseal LMA and i-gel [11–15]. The 90° rotation technique has the advantage over the previously reported methods that it does not require a separate tool and reduces pharyngeal mucosal trauma.

It is unknown whether this method increases the success rate of LMA Flexible insertion. Thus, we evaluated whether the 90° rotation technique increased the success rate of the LMA Flexible insertion compared to the standard insertion method.

## Methods

This prospective randomized study was approved by the Seoul National University Bundang Hospital Institutional Review Board (B-1409/265–005) and was registered at ClinicalTrials.gov (NCT 03028896). Written informed consent was obtained from all participants.

In total, 129 female patients aged 18–65 years, with an ASA physical class I–II and who were scheduled for elective breast surgery under general anesthesia using the LMA Flexible, were recruited to the study. Exclusion criteria were a known difficult airway, mouth opening less than 2.5 cm, limited extension of the neck, recent sore throat, and gastro-oesophageal reflux disease. Patients were randomised into two groups by a nurse who was not involved in the rest of the study using computer-generated random numbers (Random Allocation Software, ver. 2.0). Premedication with midazolam 0.03 mg kg^− 1^ I.V. was performed in the reception area. Anesthesia was induced with I.V. propofol 1.5 mg kg^− 1^, alfentanil 5 μg kg^− 1^, and rocuronium 0.5 mg kg^− 1^, and was maintained with inhaled desflurane. Insertion of the LMA Flexible was performed by a single anesthesiologist who had experience with more than 500 cases with the standard technique. A size 3 LMA Flexible for patients weighing 50–70 kg and a size 4 LMA Flexible for those weighing 70–100 kg, were used. The standard method followed the manufacturer’s instructions [16]. In the neck-flexed-and-head-extended position, holding the mask like a pen and with the index finger placed anteriorly at the junction of the cuff and tube, the mask was pushed backwards, maintaining pressure against the palate until resistance was felt. The 90° rotation method was the same as in previous reports: after insertion of the entire cuff inside the mouth, the LMA Flexible was rotated anticlockwise through 90° and advanced through the right side of the tongue until resistance was felt, and was then then turned back in the hypopharynx [11–14]. In both methods, insertion of the LMA Flexible was done with the cuff deflated, and followed by re-inflation to 60 cmH_2_O using a manometer. Effective ventilation was indexed by a square-wave capnograph trace and no audible leak during manual ventilation at peak airway pressures ≥10 cmH_2_O. If ventilation was ineffective, manipulations like jaw thrust, chin lift, and extension and flexion of the neck were allowed. If ventilation was still ineffective, re-insertion was tried up to three times. The insertion time was defined as time from mouth passage of the device to effective ventilation after inflation of the cuff. The oropharyngeal leak pressure was checked by hearing the leak sound at mouth using stethoscope while watching the pressure gauge of the ventilator and manually inflating the bag at a fresh gas flow of 5 L min^− 1^ with the pop-off valve closed. The number of insertion attempts, number of manipulations needed during insertion, and insertion time were recorded. Heart rate and mean arterial pressure (MAP) were checked 1 min before and after LMA Flexible insertion.

At the end of surgery, the LMA Flexible was removed after confirming the return of spontaneous ventilation and consciousness. A nurse blinded to the patient group checked for blood staining of the LMA Flexible after removal. Sore throat was rated on a numerical rating scale (NRS; 0–10) by asking to patients a standard questionnaire before discharge from the post-anesthetic care unit (PACU). A score of more than 4 points on the NRS was considered to indicate a sore throat.

### Statistics

The primary outcome was the success rate of first-attempt insertion of the LMA Flexible, which was confirmed by successful ventilation. Secondary outcome variables were insertion time, the number of trials and manipulations required for proper positioning, oropharyngeal leak pressure, and postoperative complications, such as blood staining of the mask and sore throat. The sample size was calculated on the basis of a previous study reporting the success rate of first-attempt insertion of the LMA Flexible to be 81% [17]. We assumed the success rate of the rotation technique would be 97% [13]. Thus, 59 patients per group would be needed to detect the difference with a power of 80% and type 1 error of 0.05. Considering a 10% drop-out rate, we recruited 66 patients per group. Patients’ characteristics, insertion time, and air leak pressure were compared using Student’s *t*-test after the Kolmogorov-Smirnov test. Success rates, blood staining of the LMA flexible, and sore throat were compared using χ^2^ and Fisher’s exact test. Repeated-measures analysis of variance was used to evaluate the effect on haemodynamic changes after LMA Flexible insertion. Data are presented as mean (± SD), median (Range), or number of patients (proportion). A *P* value < 0.05 was considered to indicate statistical significance.

## Results

**Fig. 1:**
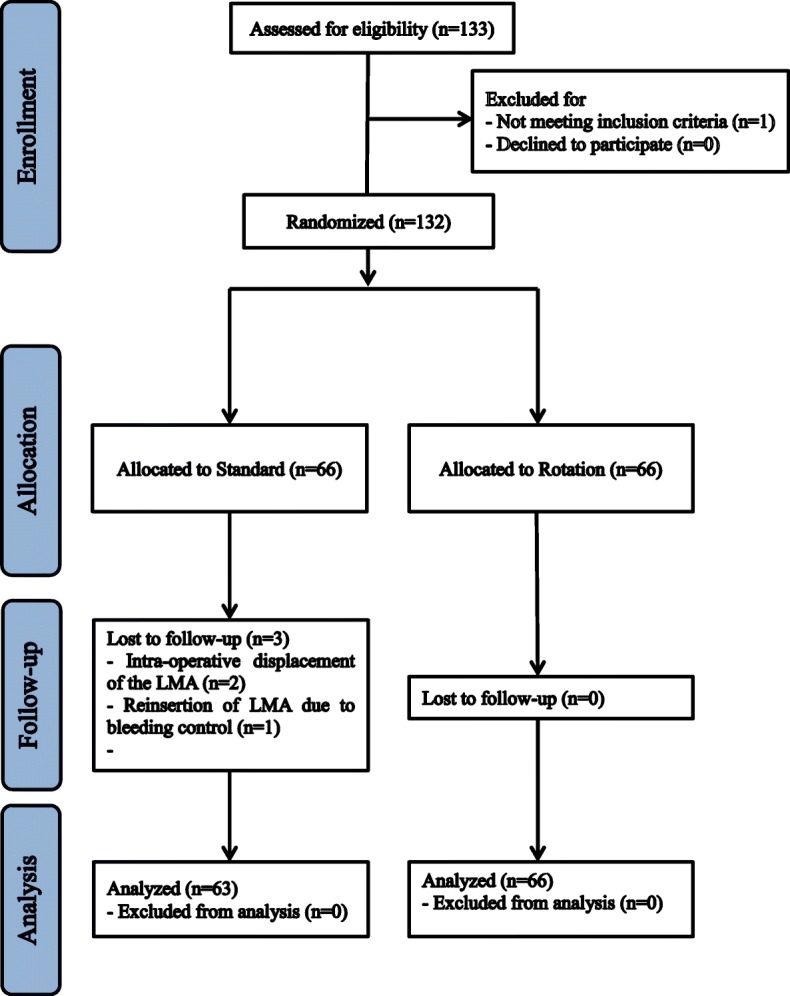
CONSORT diagram

In total, 132 patients were enrolled. One patient did not meet the inclusion criteria and three were excluded after randomisation, leaving 129 patients to be analysed. The standard and the rotation groups finally included 63 and 66 patients, respectively (Fig. 1). Patient characteristics did not differ between the groups (Table 1).

**Table 1:** Patient characteristics

	Standard (*n* = 63)	Rotation (*n* = 66)	*p-*value
Age (years)	42.1 ± 10.2	47.4 ± 11.4	0.09
Height (cm)	160 ± 6.1	159.2 ± 5.6	0.99
BMI (kg/m^2^)	22.4 ± 2.8	23.3 ± 3.5	0.57
ASA (I/II)	59/5	46/20	0.13

Values are number of patients, mean ± SD. *BMI* body mass index, *ASA* American Society of Anesthesiologists

The first-attempt success rate was not significantly different between the groups (59/63, 93.7% vs. 65/66, 98.5%, *P* = 0.20). The LMA Flexible was inserted successfully at the first attempt in almost all patients except for four in the standard group and one in the rotation group. The insertion time (10.5 ± 4.7 vs. 9.7 ± 4.7, *P* = 0.58), number of trials and manipulations required for proper positioning (2/63, 3.2% vs. 1/66, 1.5%, *P* = 0.61), and air leak pressure (19.8 ± 4.9 vs. 20.5 ± 4.2, *P* = 0.38) also showed no significant difference between the groups (Table 2). MAP and heart rate 1 min before and after the insertion of the LMA did not differ between the groups. Blood staining on the LMA Flexible after removal (1/63, 1.6% vs. 3/66, 4.5%, *P* = 0.33), and the incidence (6/63, 9.5% vs. 12/66, 18.2%, *P* = 0.21) and degree [NRS 3 [1–8] vs. 3.5 [1–7], *P* = 0.16] of sore throat checked in the PACU before discharge, were also not different between the groups (Table 3).

**Table 2:** Intraoperative variables

	Standard (*n* = 63)	Rotation (*n* = 66)	*P-*value
Success rate (n, %)			
First attempt	59 (93.7)	65 (98.5)	0.2
Second attempt	3 (4.8)	1 (1.5)	0.36
Third attempt	1 (1.6)	0 (0)	0.49
Manipulations required (n, %)	2 (3.2)	1 (1.5)	0.61
Neck extension	0	1	
push in LMA	1	0	
cuff pressure adjustment	1	0	
Insertion time (sec)	10.5 ± 4.7	9.7 ± 4.7	0.58
Air leak pressure (cmH_2_O)	19.8 ± 4.9	20.5 ± 4.2	0.38
Operation time (min)	51.7 ± 26.5	57.3 ± 29.4	0.26
Anesthesia time (min)	71.4 ± 26.8	77.8 ± 30.2	0.21

Values are number of patients (%), mean ± SD. *LMA* laryngeal mask airway

**Table 3:** Hemodynamic variables and complications

	Standard (*n* = 63)	Rotation (*n* = 66)	*P-*value
MAP (mmHg)			
Before insertion	65.5 ± 13.4	63.3 ± 10.9	0.31
After insertion	61.1 ± 12.7	63.7 ± 14.7	0.29
Heart Rate (beats/min)			
Before insertion	73.7 ± 14.3	70.4 ± 12.0	0.16
After insertion	73.0 ± 17.2	70.1 ± 11.5	0.27
Blood staining (n, %)	1 (1.6)	3 (4.5)	0.33
Sore throat at PACU (NRS)	3 (1–8)	3.5 (1–7)	0.16
Sore throat (NRS > 4) (n) %	6 (9.5)	12 (18.2)	0.21

Values are number of patients (%), mean ± SD, or median (range). *MAP* mean arterial pressure, *PACU* post-anesthetic care unit, *NRS* numerical rating scale (0–10)

## Discussion

A 90° rotation technique for insertion of LMA was first proposed by Hwang et al. for the ProSeal LMA. They showed that the rotation technique was more successful than the standard technique and was associated with less pharyngeal mucosal trauma. They explained that this was because the lateral edge of the LMA reduced resistance between the LMA and the posterior pharyngeal wall [11]. We would add that another advantage of the 90° rotation technique is that insertion of the operator’s fingers into the patient’s mouth is not necessary. The value of this advantage would be greater when the patient is awake and the possibility of biting exists. In subsequent studies, they showed that rotation technique was superior to the standard technique in various size of ProSeal LMA including children, with or without the use of neuromuscular blockers, and also in i-gel [12–14].

In this study, the first attempt success rate for insertion of the LMA Flexible was not significantly different between the two methods used. This is different from findings of previous studies reporting superiority of the 90° rotation technique compared to standard method. However, this was not because the 90° rotation technique was ineffective but because the success rate of both methods was high enough. Indeed, the first attempt success rate of rotation group was 98.5%, which is comparable to previously reported ones of 97–100%. In contrast, the first attempt success rate of 95.3% of standard group is higher than those reported previously with ProSeal LMA or i-gel. This is also higher than previously reported first-attempt success rate for the LMA Flexible of 81.5–90% [17, 18]. One study reported that use of a laryngoscope increased the first-attempt success rate from 81.5 to 96.3% [17]. There has been recognition that insertion of the LMA Flexible is more difficult than that of other LMAs, because it is difficult to transmit force along the flexible shaft [5–8]. They reported that the use of an extra-tool; such as a modified Magill forcep, a stylet, the Bosworth introducer, and the spatula introducer; would facilitate the insertion of the LMA Flexible. However, these methods not only require an extra-tool but also be a reason of trauma to the larynx. The benefits of the 90° rotation technique compared to those methods are no need for an extra-tool and decrease of laryngeal trauma. In our study, we did not find the LMA Flexible more difficult to insert versus other LMAs using either the conventional or 90° rotation technique. One reason for our relatively high success rate at the first attempt might have been the use of a neuromuscular blocker in our patients. Previous studies reported that use of neuromuscular blockers increased the pharyngeal space and improved the efficacy and success of insertion [19, 20]. Another reason may have been that we only included young female patients and excluded patients with the possibility of a difficult airway. We used an LMA that was one size smaller than the manufacturer’s recommendations and this might also have affected the first-attempt success rate. We chose this smaller-sized LMA based on previous studies showing decreased mucosal injury and postoperative sore throat with the use of a smaller-sized LMA [21, 22]. We could find a previous study reporting classic and rotatory methods being comparable in paediatric patients using classic LMA. However, they used 180° rotation which is differ from our 90° rotation method [23].

Postoperative sore throat is a common complication after general anesthesia and the reported incidence is up to 35% with use of the supraglottic airways [17, 24]. The overall incidence of sore throat in our patients was 13.9%, which was relatively low compared with previous reports. In addition, the degree of sore throat was not severe with the median (range) NRS of 3 [1–8] and 3.5 [1–7] in each groups. The most important factors for the development of sore throat after insertion into the supraglottic airways are trauma during device insertion and a high intracuff pressure. However, most of the insertions in our patients were quick and smooth, as shown by the short insertion time of around only 10 s in both groups. We also controlled the intra-cuff pressure to < 60 cmH_2_O using a manometer. These factors might be related to our relatively low incidence of postoperative sore throat. Female gender and younger age are also risk factors for postoperative sore throat and these factors might have been associated with our results, where all of our patients were relatively young female patients undergoing breast surgery [25].

This study had some limitations. First, double blinding was not possible because we could not disguise the insertion method. Second, most of our patients were relatively healthy, young, females and we excluded those with suspected difficult airways. Thus, it is unclear whether our results would generalise to the general population, including men, the elderly, and difficult airway patients. In a previous report studying more than 15,000 patients anesthetized using LMA, male sex and increased body mass index were independent risk factors for failed LMA, which was defined as and airway event requiring LMA removal and tracheal intubation [26]. The incindence of postoperative sore throat was higher in females compared to males after the use of LAM [27]. Third, a single investigator who was an expert in the use of LMA placed all LMA. This also limits generalizability of the finding of this study and additionally and might have affected the high incidence of the first attempt success rate of both methods in this study. Fourth, we used a neuromuscular blocker before insertion of the LMA Flexible and this might have affected the insertion conditions. Fifth, we did not use objective monitors such as bispectral index (BIS) monitor or nerve stimulator and cannot rule out the possibility of difference in depth of anesthesia or neuromuscular blockade among the patients. The last, we did not confirm the proper position of LMA using fiberoptic bronchoscopy and used clinical assessment of proper ventilation only. We checked the proper positioning of the LMA by a clinical indicator, such as an oropharyngeal leak pressure. If the position of the LMA is improper, it cannot seal the pharynx well, and the leakage of air will occur at lower pressure. This indicator has been widely used as an indicator of proper position of the LMA [28, 29]. However, caution is needed when comparing our data with those assessed proper positioning of LMA with direct visualization.

## Conclusions

The 90°rotation technique was as effective as the standard method for insertion of the LMA Flexible in female patients undergoing breast surgery. Complications, such as blood staining and sore throat, were also comparable. The 90°rotation technique seems to be a good alternative to the standard method when using the LMA Flexible.

## Data Availability

The full study protocol and raw data set can be obtained from Dr. Ah-Young Oh (ohahyoung@hanmail.net).

## Abbreviations

ASA: American Society of Anesthesiologists
BMI: Body mass index
IV: Intravenous
LMA: Laryngeal mask airway
MAP: Mean arterial pressure
NRS: Numerical rating scale
PACU: Post-anesthetic care unit
SD: Standard deviation
